# Immunogenicity of infectious pathogens and vaccine antigens

**DOI:** 10.1186/s12865-015-0095-y

**Published:** 2015-05-29

**Authors:** Siddhartha Mahanty, Antoine Prigent, Olivier Garraud

**Affiliations:** Laboratory of Parasitic Diseases, National Institutes of Allergy and Infectious Diseases, National Institutes of Health, Bethesda, MD 20892 USA; Etablissement Français du Sang (EFS) Auvergne-Loire, 42023 Saint-Etienne, France; EA3064, Université de Lyon, 42023 Saint-Etienne, France; Institut National de la Transfusion Sanguine (INTS), 75015 Paris, France

**Keywords:** Immunogenicity, Infection, Pathogen, Antigen presentation, Vaccine, Antibody isotype

## Abstract

The concept of the immunogenicity of an antigen is frequently encountered in the context of vaccine development, an area of intense interest currently due to the emergence or re-emergence of infectious pathogens with the potential for worldwide spread. However, the theoretical notion of immunogenicity as discussed in older textbooks of immunology needs reconsideration due to advances in our understanding of immunologic responses. Immunogenicity is a property that can either be a desirable attribute, for example in the generation of an effective protective immunity against infectious pathogens or an undesirable trait, for example when it relates to novel therapeutic compounds and drugs, where an immune response needs to be prevented or inhibited. In this Forum Article, we aimed to revisit the issue of immunogenicity to discuss a series of simple questions relevant to the concept that are frequently rephrased but incompletely resolved in the immunologic literature.

## Introductory remarks and the definition of immunogenicity

A formal definition of immunogenicity can be stated as “the ability of a molecule or substance to provoke an immune response” or “the strength or magnitude of an immune response” [1]. In this definition, the term “immune response” refers to “an integrated systemic response to an antigen (Ag), especially one mediated by lymphocytes and involving recognition of Ags by specific antibodies (Abs) or previously sensitized lymphocytes” [2]. As such, the definitions are uniquely indicative of the adaptive immune system (that gives rise to adaptive immunity, vis a vis innate immunity).

Adaptive immunity either relays innate immunity if it not succeeded in completely clearing an infection by a pathogen (thus serving as a second line of defense against the invading or damaging agent) or is mobilized in a straighter way when the body faces pathogens’ moieties to which it has already developed tools (post natural or vaccine infection). For this line of defense to succeed, certain conditions need to be satisfied: 1) instructions for engaging the non-self pathogen have to be conveyed from innate immune cells to professional or ad hoc Ag presenting cell (APCs); 2) a favorable environment has to be generated that facilitates cell recruitment and growth, and inhibits apoptosis and attrition of effector cells, not only by local tissues but also in draining lymphoid tissues such as lymph nodes to which the pathogens are transported by specialized cells [3]. Adaptive immunity principally relies on stringently recognized epitopes selected from a finite, albeit immense, library of T and B lymphocyte receptors (TcRs, BcRs). In short, an efficient adaptive immune response requires ligation of an optimally matched epitope to a complementary TcR. This process involves changes to the TcR that occur in the expressing lymphocyte as it passes through the germinal center (GC) of the draining lymph node. These changes, in turn, alter T and B cell interactions, promoting B cell maturation in the same GC via hypersomatic mutations that occur in centroblasts within the GC dark zone, and isotype commutation (in centrocytes in the GC light zone) [4]. Cell to cell communication through multiple adhesion sites and cytokine and chemokine action through their respective receptors are essential for this process, controlling the T and B cell migration and, consequently, their terminal differentiation [4]. An optimal response of this immunological reaction is the goal of vaccine candidates that, ideally, aim to reproduce responses elicited by natural, pathogen-derived peptide epitopes. Thus, immunogenicity is more or less the property of an Ag that allows the efficient fulfillment of each of the steps outlined above; if the Ag fails to trigger any of these steps, the adaptive immune response is either ineffectual or fails to develop. Steps of this process that can be modeled in human immune cells through ex vivo experiment or in animal models provide insights into a number of critical determinants of the immunogenic properties of Ags.

In general, immunogenicity may not be a factor that is a sine qua non for immunity to infection. Immunogenicity appears not to be a crucial factor or even a requirement for eliciting a robust response against pathogens and, in certain circumstances, pathogen-derived molecules, as it seems that low immunogenicity can be overcome by other factors. Conversely, immunogenicity is not a desirable property when the foreign molecule or Ag needs to be tolerated and not recognized as a pathogen. Examples of such Ags include those derived from allogeneic blood cells, transplanted hematopoietic cells, homologous tissues or solid organs or therapeutic biologics. In such situations it is often necessary to reduce Immunogenicity using immunomodulating procedures such as tolerization (in case of allergy, or unintended immunization to biologics). In the case of infectious pathogens, reduction in the immunogenicity of an Ag is generally not a desired outcome unless there is concern about cross-reacting epitopes that induce autoimmunity or where immunopathology accounts for disease manifestations.. Interestingly, in such situations, there may have to be a tradeoff between the reduction in immunogenicity and protective immune responses. This is often the case with infections with intracellular pathogens such as Mycobacterium tuberculosis Toxoplasma gondii, Plasmodium spp., and Schistosoma spp., to cite a few [5].

The concept of immunogenicity is also connected inextricably with the issue of immune memory. Immune memory, which can be generated in the central or peripheral immune system, is a hallmark of adaptive immunity and is one of the major outcomes of an effective, specific response [4]. Indicators of effective immunity include the sustained production of effector lymphocytes, and, conversely, the development of tolerance. The immunogenicity of an Ag is instrumental in directing Ag-specific lymphocytes to a dedicated response pathway. In general, the greater the immunogenicity of an Ag, the smaller the amount of Ag required to elicit an immune response, the more robust the recall responses (peripheral memory) and higher the affinity of the epitope/target interaction. Thus, our understanding of immunogenicity, as a property of an Ag, suggests that it is crucial both for determining the initial immune response to a given Ag as well as the strength and duration of the adaptive immune response over the longer term.

## Unanswered questions regarding the determinants of immunogenicity

For decades, there has been active debate about to the determinants of immunogenicity in reviews and textbooks. A search on PubMed today yields a large number of entries, but nearly all reviews discussing the factors that determine antigenic immunogenicity are focused on two aspects: 1) the requirements for development of improved vaccines against resistant microbes, and 2) the avoidance of unwanted immune response against biologics and, particularly, against therapeutic monoclonal Abs. This intense interest centers around a number of questions regarding the nature of immunogenicity that remain unanswered after more than 10 years of discussion on the determinants of immunogenicity. These questions can be simply stated as:

1. Why are some infectious pathogens weakly immunogenic?
2. What is an immunogenic or major epitope?
3. What is optimal Ag presentation?
4. What is the optimal cellular environment for facilitating and supporting Ag- reactive (previously termed “Ag-specific”) T and B cells?
5. What drives isotype switching? Why is it necessary? And what makes an Ab “protective”?
6. Why is a long term memory response less robust when elicited by engineered peptides than by wild-type pathogen-derived Ags?

## Why are some infectious pathogens so poorly immunogenic?

It is striking that although more than 130 years have elapsed since the first engineered human vaccine for a pathogen was successfully administered to a patient (and near 220 years after the perhaps “first” vaccine by Jenner); despite decades of research focused on identifying protective vaccine candidates, there are, as yet, no safe and protective vaccine for many infectious agents. Undoubtedly, the complexity of many pathogens is a major obstacle to success in this area of research. This is reflected in the fact that no practical vaccines have been developed against intracellular or extracellular parasites, although a few examples of natural vaccines are traditionally cited as successes, such as leishmanization for cutaneous leishmaniasis [6]. While the non-protein part of bacteria (especially sugars from the capsule and/or the surface) may elicit Ab responses, those are infrequently neutralizing as opposed to agglutinating, and they are not thought to originate from T-dependent B cell responses [7,8]. In these situations, T cell-independent, extrafollicular B cell responses tend to predominate with low-affinity IgM responses and no memory responses. It appears that only the peptides and toxins appear to be predictably immunogenic and induce protective responses. Mycobacteria are particularly interesting because one vaccine—Bacille Calmette-Guerin (BCG)—has been available since the late 20’s (a result of 40 years of research), but in the large clinical trials has been found to be insufficiently protective against pulmonary tuberculosis in humans [9]. Despite concerted research for almost 80 years, little progress has been made with improvement of the immunogenicity of BCG.

In contrast, viruses present a different story. Most antiviral vaccines are peptide- based, and likely as a consequence, a number of these vaccines have been more effective than those for bacterial and parasitic pathogens mentioned above [10,11]. Indeed, the success of several antiviral vaccines has raised the hope of, and in one case (variola), even succeeded in eradicating certain viral infections. Nevertheless, the same strategy that succeeded in developing effective vaccines for some viruses has failed for are many viral infections. Examples of unsuccessful vaccines include those for HIV, EBV, HSV, Dengue virus, and other emerging viral pathogens [12]. Nucleic acid-based vaccines have also been disappointing, not only for cancer and autoimmune conditions, but also for infectious diseases, for example with HIV [13]. In the case of prion-associated diseases, despite the peptide nature of prions, not only is there little progress for development of vaccines, even Ab-based tools for diagnosis have not been achieved [14]. Thus, it appears that the abundance of non- protein Ags among infectious pathogens is not the only factor that limits immunogenicity.

## What is an immunogenic versus a major epitope? How does this relate to the issue of antigenic competition?

Although there has been a remarkable lack of success in the development of vaccines against some of the major infectious pathogens, there are some salutary lessons learned from research in vaccine engineering. Repeated failures of selected vaccine candidates, selected on apparently sound immunological rationales raises questions about the nature of epitopes that induce sustained T- or B-cell responses versus their capacity to induce functionally protective effectors. Some of those epitopes are undoubtedly useful as diagnostic tools, but disappoint as vaccine candidates. As Ags, these molecules do possess qualities that would be predicted to confer immunogenicity. These epitopes are frequently expressed abundantly on the surface of infectious pathogens (often as components of envelope structures) and are composed of simple repeated motifs. Their biochemical composition (alignment and/or folding of amino acids [AA]) largely explains their capacity to stimulate T cells (via their TcR) and B cells (via their BcR or by cognate interactions with T cells). The strong immune responses resulting from stimulation by these Ags also implies that the major histocompatilibity complex (MHC) can efficiently bind those peptides and present them to reactive TcRs. Alternatively, or additionally, a large number of MHC molecules may be able to bind those peptides, implying a weak MHC restriction. However, when one tries to immunize animals with such antigenic peptides for selection of vaccine candidates or to apply those peptides to in vitro selection of hybridomas for mAbs, the cells or recipient animals produce a variety of Abs, most of which react to a limited number of irrelevant targets, often molecules that are widely distributed on cell surfaces, cytoplasm or nuclei, and are widely cross- reacting as well [15]. Consequently, induction of Abs to specific epitopes of interests is a rare or very rare event. It appears that it will be necessary to inhibit the cellular response to cross-reacting epitopes to favor the possible selection of one or a few epitopes of interest. These observations raise the question: How does competition between epitopes serve as an impediment to selection of these epitopes of functional interest? The best strategies for favoring responses of interest and inhibiting the irrelevant responses remain a large challenge, not only in immunity to infection but also against infection-associated cancers and in auto-immunity. Thus, it appears that the immunogenicity of an Ag is essential for the induction of an immune response but apparently not sufficient to achieve effective immunity.

## What is optimal Ag presentation?

As has been mentioned above, the central issue of peptide immunogenicity is Ag presentation. At a simplistic level, the capacity for an Ag to be presented to a reactive lymphocyte can be thought of as the ability of an HLA molecule to replace the “house-keeping” peptide by a short (8-20) AA sequence from the foreign Ag that matches and complements the Ag binding groove on the HLA molecule [16]. The AA sequence is immobilized within the HLA groove by physicochemical bonds and is available to bind the corresponding TcRs on T lymphocytes. Ag presentation requires export of the HLA molecule bound to the peptide to the surface of the APC surface. The constraints on Ag presentation are, thus, the presence of APCs at the site of pathogen entry, the ability of the pathogen to be degraded into AA sequences suitable for HLA binding, and the frequency of HLA molecules (types or “species”) capable of binding to the AA peptide molecule. There has been considerable progress in our understanding of the Ag presentation process since the discovery of MHC restriction, but much of our modeling of human Ag presentation derives directly from models of H2 (mouse MHC) molecules, in which Ags appear to be more stringently restricted to H2 types than perhaps occurs in humans [17]. In general, when considering vaccine Ags, the goal is to select candidate vaccine Ags that are less HLA restricted and able to bind a broad spectrum of common HLA types. In developing vaccines for infectious diseases, the goal is induce effective immunity in more individuals through Ags selected for optimal presentation, providing the best chance for a protective response to a subset of Ags. Paradoxically, Ags from infectious pathogens that are not well suited for peptide degradation and for Ag presentation possess a selective advantage allowing their escape from immune surveillance [18].

In contrast to vaccines, when foreign tissues are injected or implanted into humans for therapy (blood, cells, organs, biologics) the goal is to avoid accidental binding of donor-derived Ags with recipient HLA molecules that subsequently present to T or B cells and induce an immune response resulting in an undesirable alloimmunization and rejection [19]. In this setting, strength of immunogenicity is inversely related to therapeutic utility, and the principles applied to the selection of antigenic targets are the converse of those described above for infectious pathogens, The questions raised above regarding the crucial role of Ag presentation as a determinant of immunogenicity are only a few of many unresolved issues in our understanding of immunogenicity. A better understanding of these determinants of immunogenicity will undoubtedly facilitate the translation of rational immunologic approaches to vaccinology and transplantation.

## What is the optimal cellular environment for facilitating and supporting Ag-reactive (formerly named specific) T and B cells?

Epitope recognition and ligation of an antigenic determinant to the specific TcR is the defining step of the adaptive immune response, since this step licenses the epitope as an “Ag”, but it is not, in itself, sufficient to trigger an immune response. A number of downstream events, such as APC/T cell co-ligation, formation of the immunological synapse, immune effector cell activation and differentiation are equally necessary for the immune response to proceed. Two other sets of molecules play crucial roles: 1) cell adhesion molecules and 2) soluble cytokines. Adhesion molecules are expressed in a restricted manner on APCs and T cells and are required, partly because the tissue environment does not favor optimal immunological synapse formation. Cytokines have diverse functions in autocrine, paracrine and exocrine pathways. This may be the reason that the route of vaccines is so important [20].

The immunological environment in which the Ag interacts with immune cells appears to be particularly critical in mucosal immune responses [21,22]. There are four major issues that have become apparent in the context of mucosal immunity: the route of entry for an infectious agent; competition with or ignorance of the microbiota (the local microbial flora) in the environment; the scope of local physiological inflammation; and local immune system tools available to the host to respond to the invading Ag, such as the diffuse and organized secondary lymphoid tissues and, in the context of a chronic inflammatory response, tertiary lymphoid tissues [23]. Overall, the balance between cytokines and other immune mediators influence the outcome of the T cell activation and differentiation program. This response, in turn, enhances or inhibits the intrinsic immunogenicity of the stimulus. Although our understanding of the modes of action of many regulatory cytokines has advanced considerably in recent decades, much needs to be learned about the differences in cytokine environments induced by different classes of Ags and in different diseases, and how the environment affects the efficiency and strength of the resulting immune response.

## What drives isotype switching? Why is it necessary? And what makes an antibody “protective”?

For decades it has been known that the efficiency of Abs is affected by the nature of the constituent immunoglobulin (Ig) of the Ab. There are principally two reasons for this conclusion. Firstly, the nature of the Ag alters the heavy (H) chain class/subclass of the Ig that is induced in responding B cells. Investigations using parasite Ags revealed the role of Ag in biasing the formation of the induced Abs into distinct subclasses, in a non-random manner [24]. The role of epitopes in clonal imprinting of Ab responses has been addressed by analysis of the light (L) chains in malaria and HIV infections [25, 26], but further elucidation of the phenomenon in other infections is needed for an understanding of how generalizable these observations are to other classes of Ags and infectious diseases. Secondly, the structure of Ig (H chain/class and subclass) influences not only the biological properties of the Ab such as the capacity to bind FcRs and CRs, etc., but also the ability of the Ig/Ab to bind targets (i.e. the antigenic epitopes) [27]. Indeed, the number of H domains, S-S bonds and glycosylation affect the conformation to the Ig backbone making it, for example, more flexible (IgG3) or rigid (IgG1). In addition, Ig conformation also influences the half-life, distribution, and vulnerability of an Ab to catabolic degradation. Studies in malaria-infected populations revealed that IgG1 (Igs with a long half-life) were less efficient than IgG3 (with a short half-life) targeting the very same epitopes [28]. Cytokines and environment also critically influence the nature of the H chain isotype.

Unfortunately, little knowledge has been added in recent years to what was known 10 years ago in this area of immunology. Of note, little is known regarding the influence of the L chains on the strength of the immune response. What we know suggests that strategies to enhance or favor IgG3 responses to a peptide Ag rather than IgG1 would likely be preferable for Ags with higher immunogenicity. However, to date, it has been particularly difficult to engineer IgG3 responses in a vaccine setting [29]. Glycosylation of the Fc fragment of antibodies is also likely to play an important role in Ab-associated immunity, possibly in determining whether the responses are protective or pathogenic [30]. This is, therefore, an area of current interest and investigation because of the probable role in antibodies function (through complement activation and FcR affinity).

In addition to MHC restriction, that has been discussed above, variations in other genes are also likely to influence the immune response to Ags as well as Ag presentation (for example, with Plasmodium Ags [31]); however, much remains unknown about which classes of genes affect the nature of immune responses to pathogens and vaccine Ags.

## Why is a long-term memory response less robust when elicited by engineered peptides than by wild-type pathogen-derived Antigens?

Recent epidemiologic studies reveal several of the vaccines used in the expanded program on immunization (EPI, designed by the World Health Organization) require a boost during the teenage years or in young adulthood because of waning long term immunity. Examples of such vaccines include measles and pertussis [32]. One explanation proposed for these observations is that, with the successful application of measles or pertussis vaccination programs, establishment of herd immunity is no longer occurring by natural boosting of immunity from exposure to circulating pathogens that were prevalent in unimmunized populations. In fact, declining protection rates are significant enough to have spurred a call for a revision of the vaccination schedule, adding booster immunizations during the teen years or young adulthood. Boosts in immunity using re-vaccination is an established strategy to maintain immunity for several commonly used vaccines (e.g. tetanus toxoid, in which case immunization is recommended every 10 years), particularly where sufficient coverage of the population has been achieved to prevent exposure to natural infections. These observations regarding the decline in immunity over time have focused attention on the nature of long-term immunity to vaccine Ags, and prompted debate and reconsideration of optimal schedules for routine immunizations. In short, these observations raise important questions about the immunogenicity of synthetic vaccines in contrast to complex, multi-epitope vaccines that have been traditionally derived from inactivated pathogens or attenuated wild- type infectious agents. The failure of synthetic vaccines to generate sustained memory implies suboptimal immunogenicity and highlights the need for better understanding of immunogenicity in relation of Ag configuration or formulation.

## Is there any other business?

This short review has essentially addressed the issue of the quality of the Ab response, and not much its quantitative related issues, apart the possible overstimulation of the immune system—the extrafollicular pathway—by non- protein Ags, leading to the production of polyreactive Abs, that often useful for assisting if not clearing an infection [33]. The issue of excess of reactant in the Ag/Ab complex relates to a very old debate in immunology, with the prozone effect; it is presently revived because of newer tests that are complicated by this phenomenon [34]. This enlightens the issue that Ags that elicit very strong Ab responses will not lead to protection through the presence of too much specific Ag (as seen in malaria, for example [35]).

Further—though this exceeds the present topics—novel approaches are urgently needed to increase immunogenicity of novel vaccine candidates. Indeed, ways to improve already existing vaccines or to “create” new vaccines encompass: -i) the vaccinated own parameters (capacity to raise a suitable response, meaning that the immune system is mature enough and the nutritional status correct); ii) the nature of the epitopes (chemistry) plus the route, the presentation and the circumvention of the HLA restriction, the adjuvanticity or the delivery vehicles, etc. The genome sequencing era, the existence of microarrays, and the availability of new animal models allowed to revisit the search for vaccine candidates, that lead to protective Abs. However, the characteristics of long-lasting, protective responses have evolved overtime along with the identification of novel targets for Ags and the revisit of correlates for protection. This challenge is vivid in several fields and particularly in the quest for vaccines against parasitic infections such as malaria [36].

## Concluding remarks

In summary, immunogenicity is central to the acquisition and maintenance of natural immunity to infectious agents. In this context, natural immunity refers to the immune state resulting from natural infections vis a vis immunization with synthetic Ags that mimic pathogen-derived Ags. Immunogenicity is also crucial for acquisition and maintenance of long-lived and effective post-vaccine immunity to infectious pathogens. However, if immunogenicity is a property of Ags directly associated with the pathogen, the ability to engineer epitopes has provided tools to enhance or inhibit the immunogenicity of these natural Ags to tailor the response as desired or appropriate to the immunological context. To achieve the desired alteration of responses it will be essential to gain better understanding of the determinants of immunogenicity. Recent advances in our knowledge about Ag structure and processing have given us some insight in to these determinants. These investigations have lead us to the realization that, not surprisingly, in addition to properties of the Ags themselves, these determinants include host factors that establish the optimal tissue and lymph node environments for efficient activation of the innate and adaptive immune response. However, the mechanisms by which the determinants confer immunogenicity remain to be elucidated. Knowledge of these mechanisms is particularly important in the field of vaccinology. Vaccine candidates are selected on the basis of being major epitopes that elicit a robust, protective, immune response. To identify such epitopes it will be necessary to understand the determinants of an optimal immunological response. Thus a better understanding of the nature of immunogenicity will be essential for us to adopt rational approaches to the design of vaccines for cancers and for the major infectious disease.
